# MiR-363 suppresses cell migration, invasion, and epithelial-mesenchymal transition of osteosarcoma by binding to NOB1

**DOI:** 10.1186/s12957-020-01859-y

**Published:** 2020-05-01

**Authors:** Yongtao Zhang, Fang Wang, Lina Wang, Quanbin Zhang

**Affiliations:** 1 Department of Orthopedics and Traumatology, Zibo Hospital of Integrated Traditional Chinese and Western Medicine, Zibo, 255000 Shandong Province China; 2 CT Imaging Department of Zibo Central Hospital, Zibo, 255000 Shandong Province China; 3 Laboratory of the Fifth People’s Hospital of Zibo, Shandong Province, Zibo, 255000 China; 4 Department of Orthopaedic Trauma, Zibo Central Hospital, No. 54 Gongqingtuan Road, Zibo, 255000 Shandong Province China

**Keywords:** Osteosarcoma, miR-363, NOB1, EMT, Migration, Invasion

## Abstract

**Background:**

Osteosarcoma (OS) is a primary malignant bone tumor with a high rate of metastasis and a short 5-year survival rate. MiR-363 was downregulated in a variety of tumors and played a role in suppressing tumors. However, the roles of miR-363 in osteosarcoma remain unknown; thus, the purpose of this study was to explore the functions of miR-363 in osteosarcoma.

**Methods:**

CCK-8 and transwell assays were performed to evaluate the proliferation, migration, and invasion abilities of MG63 cells. The epithelial-mesenchymal transition (EMT) and apoptosis-associated proteins were measured by using Western blot assay. Luciferase reporter assay was utilized to verify whether miR-363 directly bound to the 3′-UTR of NOB1 mRNA.

**Results:**

MiR-363 was downregulated while NOB1 was upregulated in osteosarcoma clinical tissue specimens and cell lines as compared with the adjacent normal tissue specimens and normal cell line. The miR-363 is reversely correlated with the expression of NOB1 in osteosarcoma tissues. Overexpression of miR-363 suppressed the ability of cell migration, invasion, and EMT, whereas low expression of miR-363 promoted this ability. In addition, miR-363 inhibited osteosarcoma proliferation both in vitro and in vivo and inhibited the apoptosis in MG63 cells. Interference of NOB1 could inhibit the migration, invasion, and EMT of osteosarcoma cell line MG63. NOB1 was verified to be a direct target of miR-363 and its expression was mediated by miR-363. Re-expression of NOB1 could partially reverse the inhibitory effect of miR-363 on cell migration and invasion. In addition, low expression of miR-363 or overexpression of NOB1 predicted poor prognosis of osteosarcoma patients.

**Conclusion:**

MiR-363 inhibited osteosarcoma the proliferation, migration, invasion, and EMT and induced the apoptosis by directly targeting NOB1 in MG63 cells. The newly identified miR-363/NOB1 axis provides novel insights into the pathogenesis of osteosarcoma.

## Introduction

Osteosarcoma was a primary malignant bone tumor with morbidity of 4,000,000 annually [1]. Osteosarcoma has a high metastasis that more than 80% of patients may have recurrent metastasis and the 5-year survival rate is only about 50% [2, 3]. Therefore, the identification of new molecular biomarkers involving osteosarcoma metastasis and the possible therapeutic targets for the treatment is urgently needed.

MicroRNAs (miRNAs) are endogenous non-coding RNAs of 22–28 nucleotides in length that could modulate gene expression by binding to their 3′-untranslated regions (UTR) at the post-transcriptional level [4, 5]. Increasing evidences demonstrated that miRNAs may play important functions in tumorigenesis and tumor progression [6, 7]. MiR-363 played a role in suppressing cancer in variety of tumors that include gastric cancer, papillary thyroid carcinoma, hepatocellular carcinoma, and lung adenocarcinoma [8–11]. In gastric cancer, Song et al. have discovered that miR-363 acted as a tumor suppressor to inhibit cell growth and migration [12]. Wang et al. demonstrated similar findings; miR-363 inhibited lung adenocarcinoma cell proliferation, colony formation, and tumor growth [13]. What is more, miR-363 inhibited the EMT and suppressed metastasis of colorectal cancer [14]. miR-363 inhibited cell vitality, proliferation, and colony formation ability, but promoted cell apoptosis and G1/S arrest in osteosarcoma [15]. However, in osteosarcoma, there are little papers which study on metastasis of miR-363; therefore, we now investigate whether miR-363 suppressed cell migration, invasion, and EMT in osteosarcoma.

NIN1 (RPN12) binding protein 1 homolog (NOB1) is located on chromosome 16q22.1 and consists of nine exons and eight introns [16]. The RNA substrate containing the D site of pro-ribosomal RNA is efficiently cleaved by NOB1 in a manganese-dependent manner, thereby regulating protease function and RNA metabolism [17]. NOB1 was upregulated and acted as an oncogene in a variety of cancers including cervical cancer, gastric cancer, epithelial ovarian cancer, and non-small cell lung cancer [18–21]. In laryngeal cancer, Gao et al. have discovered that silencing of NOB1 could inhibit cell growth and metastasis [22]. Dai et al. discovered a similar finding that knocking down NOB1 inhibited the proliferation and migration of colorectal cancer cells [23]. In addition, knockdown of NOB1 suppressed the malignant transformation of prostate cancer [24]. Thus, we strongly believe that knocking down NOB1 could suppress the cellular progression of osteosarcoma. The purpose of this study was to investigate the functions of miR-363 and the relationship between the expression of miR-363 and NOB1 in osteosarcoma tissue specimens.

## Material and methods

### Patients and clinical samples

From January 2014 and December 2016, patients underwent surgery at the Zibo Hospital of Integrated Traditional Chinese and Western Medicine and obtained 49 pairs of osteosarcoma and corresponding paracancerous tissues. Before surgery, no patients had received chemotherapy or radiotherapy. Two pathologists performed the pathological diagnosis of osteosarcoma. Instantly after surgery, fresh tissues were frozen in liquid nitrogen and stored at − 80 °C incubator. All the specimens of this study have got informed consent from patients and have been approved by the Ethical Committee of Zibo Hospital of Integrated Traditional Chinese and Western Medicine. The entire investigation complies with the principles outlined in the Helsinki Declaration.

### Cell lines and culture condition

All cells, including two human osteosarcoma cell lines MG63 and SaOS2 and a normal osteoblast cell line NHOst, were obtained from American Type Culture Collection (ATCC; Rockville, USA). All the cells were cultured in RPMI-1640 (Gibco, Carlsbad, USA) supplemented with 10% fetal bovine serum (FBS; Gibco), 100 IU/mL penicillin, and 100 mg/mL streptomycin in an incubator at 37 °C and a humid atmosphere of 5% CO_2_.

### RNA isolation and quantitative real-time polymerase chain reaction (qRT-PCR)

Total RNAs were extracted using the TRIzol® reagent (Invitrogen, Carlsbad, USA). To synthesize the cDNA chain, the PrimeScript™ Reverse Transcription Reagent Kit (TaKaRa Bio, Otsu, Japan) was employed to perform the reverse transcription. Step One Plus™ real-time PCR system (Applied Biosystems, Foster City, CA) and SYBR® Premix Ex Taq™ II (TaKaRa Bio) were utilized to carry out the PCR assay. The 2^−ΔΔCt^ method was applied to calculate the relative quantities of each gene. The primers were miR-363 F: 5′-GCGGCCAATTGCACGGTAT-3′, R: 5′-GTGCAGGGTCCGAGGTATTC-3′; U6 F: 5′-CTCGCTTCGGCAGCACA-3′ R: 5′-AACGCTTCACGAATTTGCGT-3′; NOB1 F: 5′-ATCTGCCCTACAAGCCTAAAC-3, R: 5′-TCCTCCTCCTCCTCCTCAC-3′; GAPDH F: 5′-GCACCGTCAAGGCTGAGAAC-3′, R: 5′-ATGGTGGTGAAGACGCCAGT-3′.

### Protein extraction and Western blotting

Total proteins were lysed and extracted by using radioimmunoprecipitation assay (RIPA) lysis buffer (Beyotime, Shanghai, China) covered with 1% phenlymethanesulfonyl fluoride (PMSF). Equal amounts of protein from each extract were separated by a 10% sodium dodecyl sulfate-polyacrylamide gel electrophoresis (SDS-PAGE). Subsequently, the proteins were transferred onto a polyvinylidene fluoride (PVDF) membrane (Millipore, Billerica, MA). Then, the membranes were incubated overnight at 4 °C with primary antibodies. The primary antibodies were against NOB1 (1:1000, Abnova, Wuhan, China), E-Cadherin (1:1000, Abcam, Cambridge, USA), and N-Cadherin (1:1000, Abcam), which were diluted in TBST (tris-buffered saline Tween). The goat IgG conjugated with horseradish peroxidase (HRP) (1:4000, Beyotime, Haimen, China) was then utilized to incubate the membranes at room temperature for 1 h. Enhanced chemiluminescence detection reagent (ECL, Beyotime) was applied to detect the signals.

### Transwell assay

Transwell chambers were inserted into a 24-well plate coated with or without Matrigel (BD Biosciences). The cells were digested with trypsin and then re-suspended in serum-free RPMI-1640 medium. Two hundred microliters of a cell suspension with 1 × 104 cells was added to the upper chamber, while 500 μl of medium containing 10% FBS was added to the lower chamber to serve as a chemoattractant. After incubating at 37 °C for 48 h under 5% CO_2_, the residual cells were removed by cotton swabs. And the migrated and invaded cells were fixed and stained with methanol and crystal violet and counted in 5 random fields using a microscope.

### Vectors and transfection

MiR-363 mimic, inhibitor, siRNA-NOB1, pcDNA3.1-NOB1, and their negative control oligonucleotide (NC) were purchased from RiboBio (Guangzhou, China). The cells were seeded into 6-well plate and incubated overnight at 37 °C. Prior to transfection, the cells were replaced with fresh medium. The vectors and Lipofectamine 2000 (Invitrogen, Carlsbad, CA) were diluted with Opti-MEM (Gibco, Carlsbad, USA) and then mixed. The mixture was added to cells in the 6-well plate and shook slowly. The cells were harvested 48 h after transfection.

### Cell counting kit-8 assay

MG63 cells (2 × 104 cells per well) were seeded on 96-well plates. After incubation for 24 h, MG63 cells were co-transfected with the miR-363 mimic or control mimic. After transfection of 48 h, the proliferation of MG63 cells was determined using the cell counting kit-8 (CCK-8) (Beyotime Institute of Biotechnology) according to the manufacturer’s instructions. The absorbance was measured with a Bio-Tek Synergy 2 microplate reader at a wavelength of 450 nm.

### Plasmid construction and luciferase reporter assay

TargetScan (http://www.targetscan.org) was applied to predict the potential target genes of miR-363, and NOB1 was discovered to be one of them. Then, the binding sequences were mutated from GUGCAAU (wild type, WT) to GACGGAA (mutant, MUT). Both the wild type and mutant NOB1 were then cloned into the pmirGlo vector (Promega, Madison, WI), and the empty vector was used as the control. After 48 h of incubation, the mR-363 mimic and wild type and mutant NOB1 were co-transfected into MG63 cells, and the luciferase reporter gene activity was calculated. Dual-Luciferase Reporter Assay System (Promega) was performed to calculate the luciferase activity ability.

### Cell apoptosis assay

FITC Annexin V Apoptosis Detection Kit I (BD Pharmingen, Franklin Lakes, NJ, USA) was used to measure the rate of apoptosis. MG63 cells (5 × 105 cells per well) were seeded into 6-well plates. After the cells are completely attached, miR-363 mimic (50 nM) or NC mimic (50 nM) is transfected into the cells. After 48 h of incubation, cells were collected, resuspended in 1× binding buffer, and incubated with 5 μL of fluorescein isothiocyanate-conjugated Annexin V and 5 μL of PI at 25 °C for 15 min in the dark. Flow cytometry analysis was performed within 1 h.

### Xenografted tumor model

Four-year-old nude mice were purchased from the Institute of Model Animals of Nanjing University. 5 × 106 cells were injected subcutaneously into the right abdomen of each nude mouse. Subsequently, when there is a significant tumor, the tumor size is measured every 3 days. After cultivating for 1 month, nude mice were sacrificed and tumors were isolated and weighed. Tumor volume was calculated by the following formula: (length × width 2)/2.

### Statistical analysis

Statistical analyses were performed using SPSS 16.0 software (SPSS, Inc., Chicago, USA) and GraphPad Prism 6.0 (La Jolla, CA, USA). All quantitative values are expressed as the mean ± standard deviation (SD). The differences between groups were compared using the Student’s *t* test and nonparametric test (Mann-Whitney *U* tests). The survival of osteosarcoma patients was evaluated by Kaplan-Meier method and the log-rank test. All other results are representative of three independent experiments. *P* < 0.05 was considered statistically significant.

## Results

### The correlation between miR-363 and NOB1 in osteosarcoma tissues

**Fig. 1:**
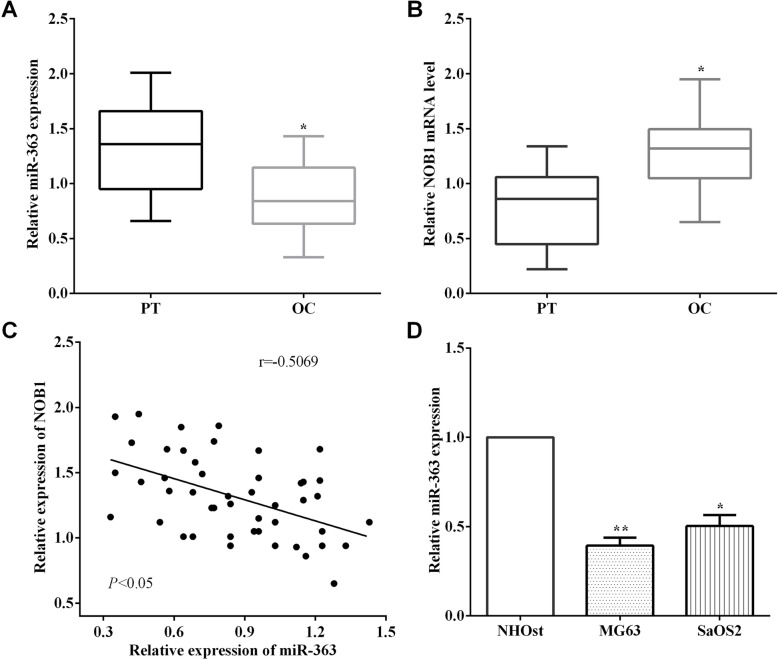
The correlation of miR-363 and NOB1 in osteosarcoma tissues. **a** It demonstrated that the levels of miR-363 were lower in osteosarcoma tissues than corresponding adjacent tissues. **b** The level of NOB1 of osteosarcoma tissues was higher than that of corresponding adjacent tissues. **c** The expression of MiR-363 and NOB1 had an inverse correction in osteosarcoma tissues. **d** The expressions of miR-363 in osteosarcoma cell lines MG63 and SaOS2 were lower than that of normal osteoblast cell NHOst. **P < 0.01; ***P < 0.001; PT, paracancerous tissues; OS, osteosarcoma

qRT-PCR was performed to measure the expression of miR-363 in 49 pairs of osteosarcoma tissue specimens and corresponding adjacent tissue specimens. As expected, it demonstrated that the levels of miR-363 in osteosarcoma tissues are lower than the corresponding adjacent tissues (*P* < 0.05) (Fig. 1a). Meanwhile, the mRNA level of NOB1 was calculated in osteosarcoma tissue specimens and the corresponding adjacent tissues. In contrast to the expression of miR-363, NOB1 showed a higher mRNA level in osteosarcoma tissues than the corresponding adjacent tissues (*P* < 0.05) (Fig. 1b). Therefore, the relationship between the expression of miR-363 and NOB1 was evaluated, and it was found to have an inverse correlation between miR-363 and NOB1 in osteosarcoma (*P* < 0.05, *r* = − 0.5069) (Fig. 1c). What’s more, the expression of miR-363 was calculated in osteosarcoma cell lines MG63 and SaOS2 and normal osteoblast cell line NHOst, and it was observed that the expression of miR-363 was lower in osteosarcoma cell lines MG63 (*P* < 0.01) and SaOS2 (*P* < 0.05) than NHOst cells (Fig. 1d).

### MiR-363 inhibited the migration, invasion, and EMT of osteosarcoma cells

**Fig. 2:**
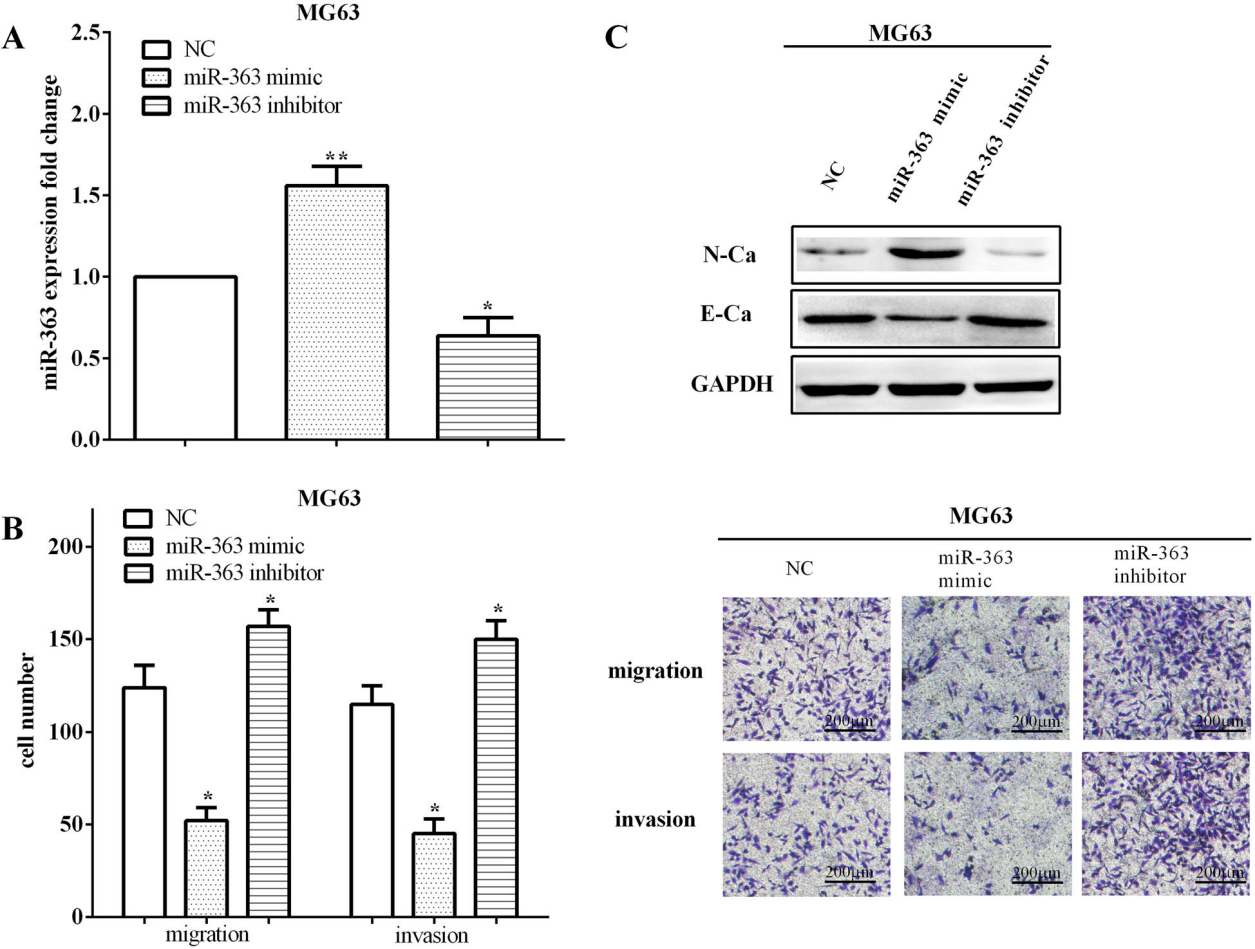
MiR-363 inhibited migration, invasion, and EMT of osteosarcoma cells. **a** MiR-363 mimic and inhibiter were transfected into MG63 cells to up- or downregulate the expression of miR-363. **b** MiR-363 mimic inhibited the abilities of migration and invasion, whereas miR-363 inhibitor increased the migratory and invasive capacities in MG63 cells. **c** Overexpression of miR-363 inhibited the EMT while low expression of miR-363 promoted the EMT of MG63 cell. E-Ca, E-cadherin, N-Ca, N-cadherin. *P < 0.05; **P < 0.01; ***P < 0.001

To illuminate the metastatic functions of miR-363 in osteosarcoma, miR-363 mimic and inhibitor were transfected into MG63 cells to upregulate (*P* < 0.01) or downregulate (*P* < 0.05) the expression of miR-363 (Fig. 2a). By calculating migratory and invasive abilities, we discovered that the miR-363 mimic inhibited the abilities of migration (*P* < 0.05) and invasion (*P* < 0.05). In contrast, the migratory (*P* < 0.05) and invasive (*P* < 0.05) capacities were increased when transfected with the miR-363 inhibitor in MG63 cells (Fig. 2b).

The EMT is an evolutionarily conservative developmental process that has been proved to be a main contributor to the metastasis of tumor. Therefore, in order to further verify the role of miR-363 in osteosarcoma cell metastasis, the differences of EMT markers were calculated when exogenous changed the expression of miR-363. As results, transfection of miR-363 mimic inhibited the EMT by improving the expression of N-cadherin, while decreasing the expression of E-cadherin. On the contrary, knockdown of miR-363 reduced the expression of N-cadherin and promoted the expression of E-cadherin (Fig. 2c), elucidating that silencing miR-363 promoted the EMT of osteosarcoma cells.

### MiR-363 inhibited tumor proliferation in vivo and in vitro and induced cell apoptosis

**Fig. 3:**
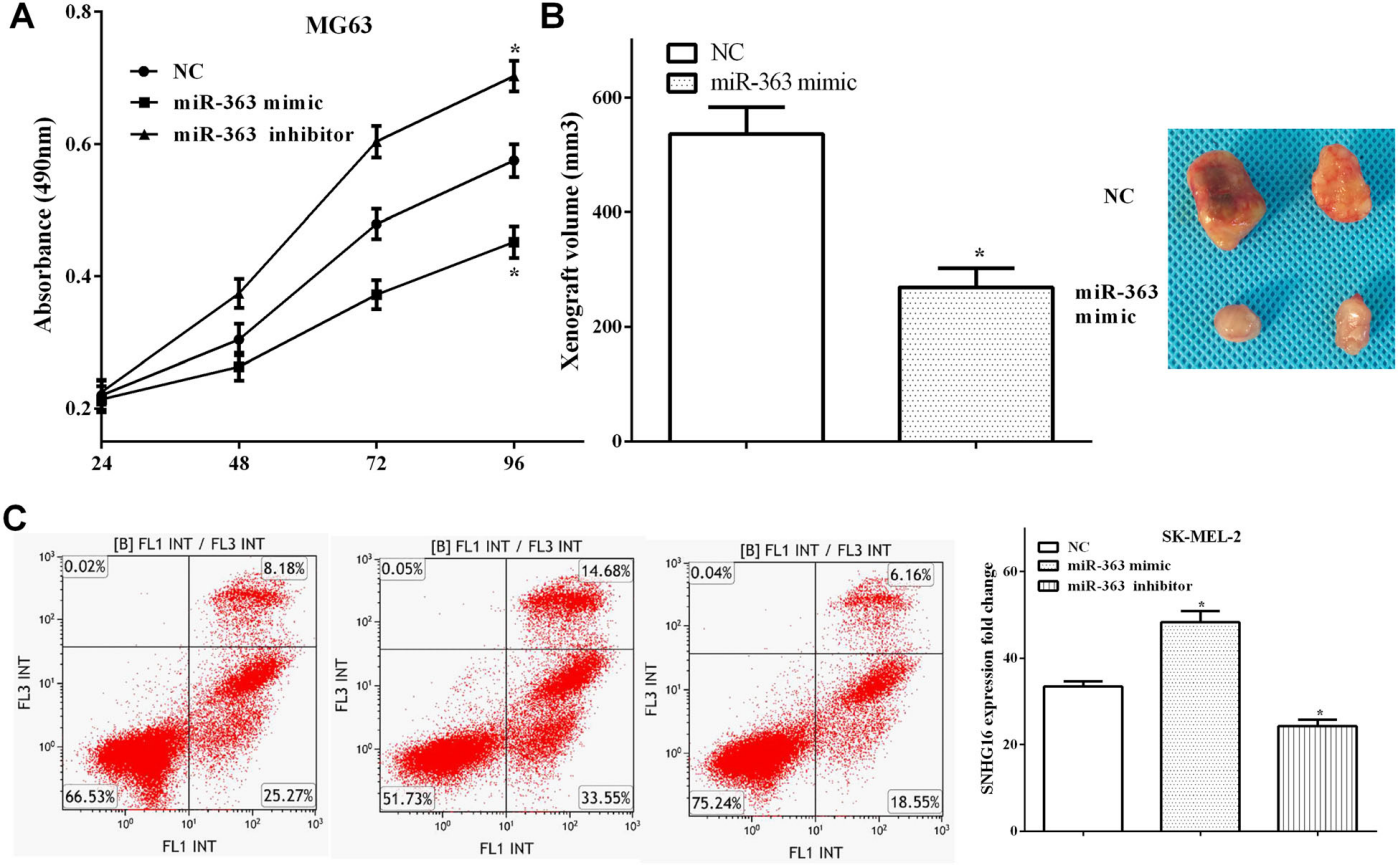
MiR-363 inhibited tumor proliferation in vivo and in vitro and induced cell apoptosis. **a** Overexpression of miR-363 inhibited cell proliferation, while cell proliferation was promoted by knocking down miR-363. **b** The volume of transplanted tumors in miR-363 overexpression group was significantly larger than that in the control group. **c** Upregulation of miR-363 induced cell apoptosis, whereas downregulation of miR-363 inhibited cell apoptosis

CCK-8 and xenograft experiment assays were performed to calculate the tumor growth in vivo and in vitro. As we expected, overexpression of miR-363 inhibited cell proliferation (*P* < 0.05), while knocking down miR-363 promoted cell proliferation (*P* < 0.05) (Fig. 3a). Similarly, the volume of transplanted tumors in miR-363 overexpression group was significantly larger than that of the control group (*P* < 0.05) (Fig. 3b). Flow cytometry analysis revealed that upregulation of miR-363 induced cell apoptosis (*P* < 0.05), whereas downregulation of miR-363 inhibited cell apoptosis (*P* < 0.05) (Fig. 3c).

### Knockdown of NOB1 inhibited MG63 cell migration, invasion, and EMT

**Fig. 4:**
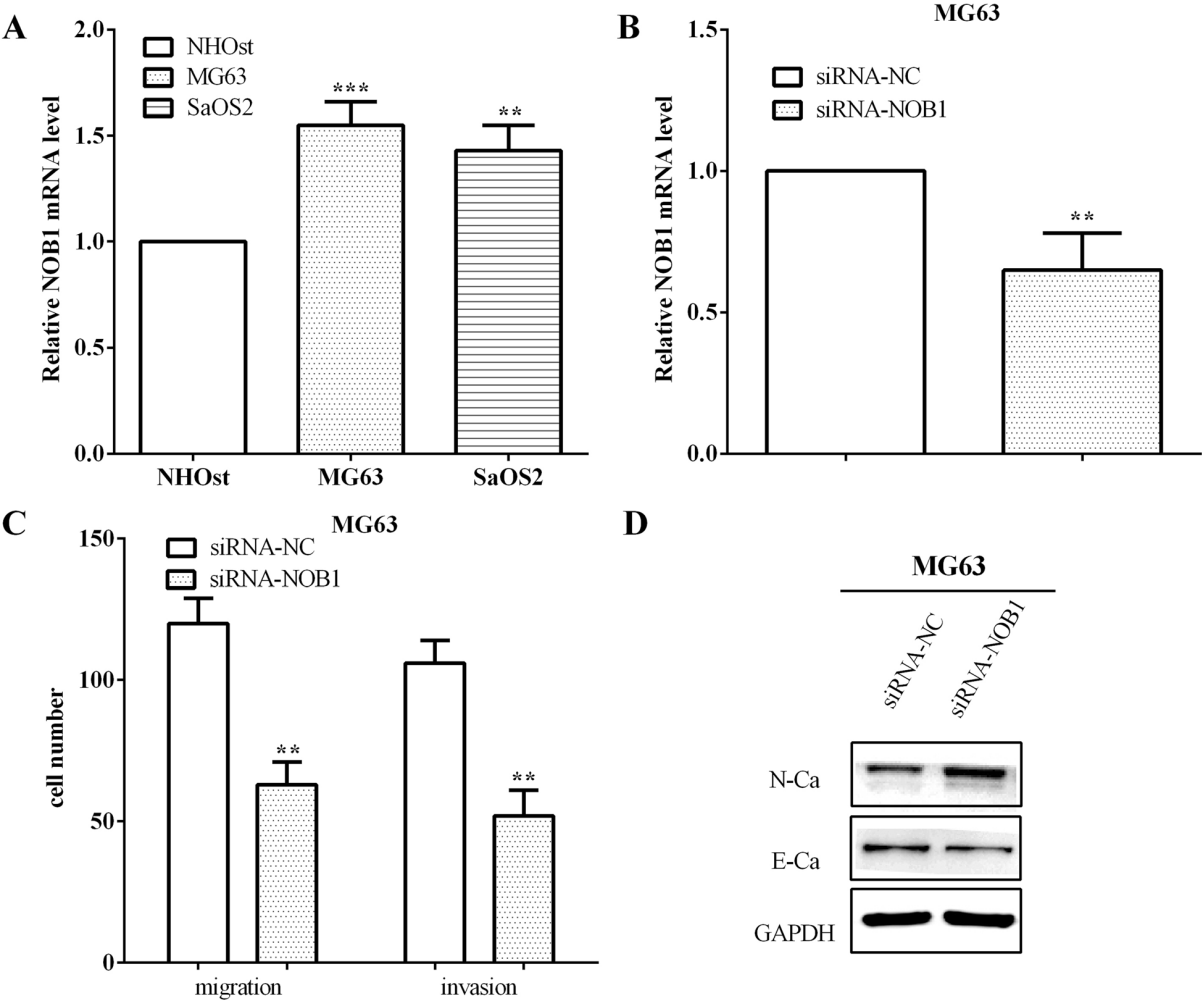
Knockdown NOB1 inhibited the migration, invasion, and EMT. **a** The expression of NOB1 was higher in osteosarcoma cell lines MG63 and SaOS2 than that of normal osteoblast cell NHOst. **b** siRNA-NOB1 was applied to interfere the expression of NOB1 in MG63. **c** The migration and invasion were decreased in NOB1 low expressed cells. **d** Interference of NOB1 could decrease the EMT of MG63 cells. **P < 0.01; ***P < 0.001

The mRNA level of NOB1 was calculated in a normal osteoblast cell line and two osteosarcoma cell lines. As expected, the results showed that NOB1 was higher in osteosarcoma cells MG63 (*P* < 0.05) and SaOS2 (*P* < 0.05) than the normal osteoblast cell NHOst (Fig. 4a). siRNA-NOB1 was applied to interfere with the expression of NOB1 in MG63 cells (*P* < 0.05), as shown in Fig. 4b. Then, the migratory and invasive ability of MG63 cells were evaluated, and it was discovered that the migration (*P* < 0.05) and invasion (*P* < 0.05) were decreased in NOB1 low expressed MG63 cells (Fig. 4c).

To further explore the impact of NOB1 on cell metastasis, the expression of EMT markers were evaluated. In MG63 cells, the expression of the mesenchymal marker N-cadherin was improved, whereas the expression of the marker epithelial E-cadherin was decreased, which has similar effects with miR-363 overexpression (Fig. 4d). All the results demonstrated that low expression of NOB1 inhibited the migration, invasion, and EMT of osteosarcoma cells, which were consistent with the results when miR-363 was overexpressed.

### miR-363 directly targeted NOB1 and mediated its expression

**Fig. 5:**
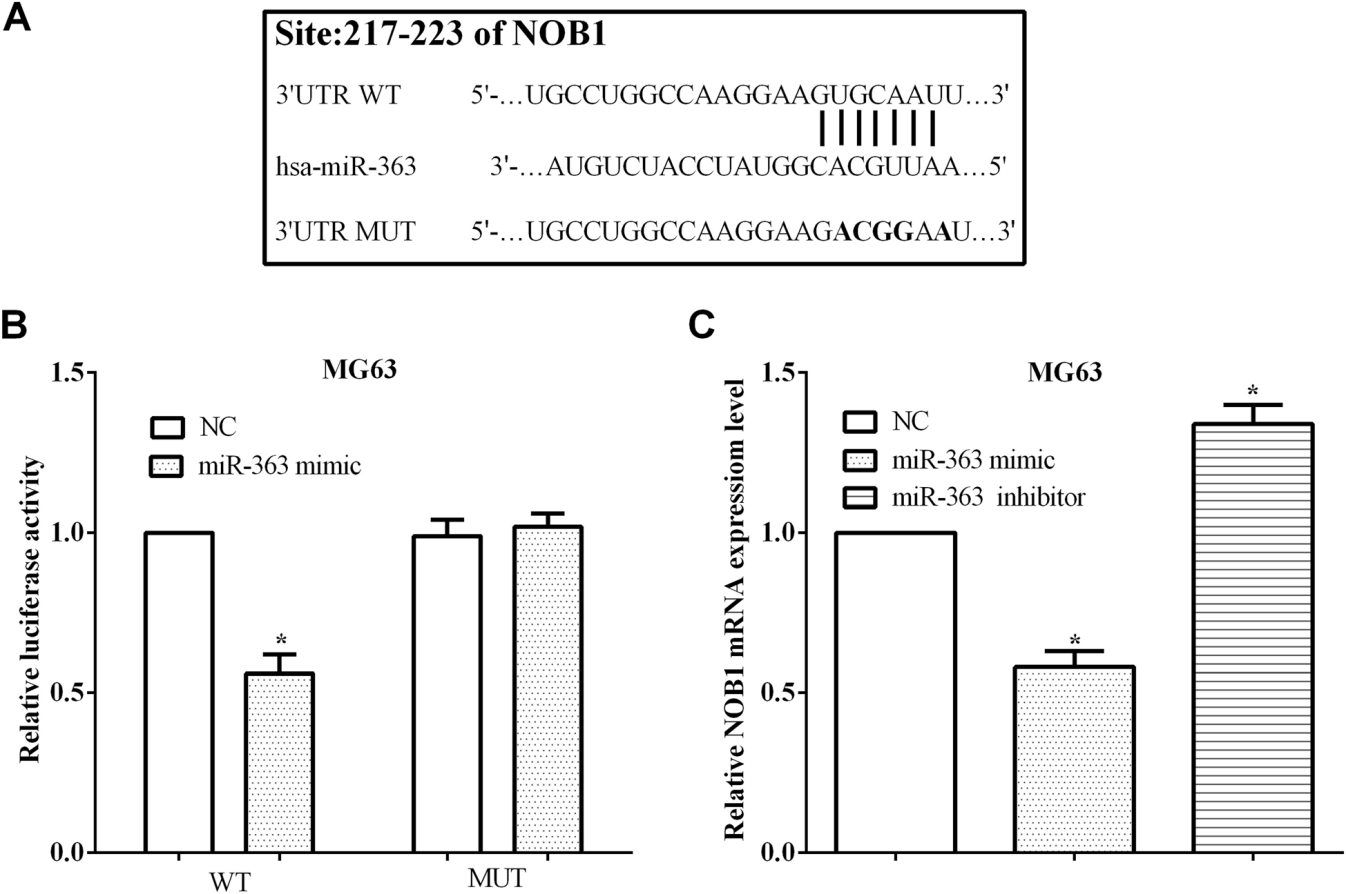
MiR-363 targeted NOB1 and mediated its expression. **a** The wild type (WT) and mutant (MUT) sequences on the 3′-UTR of NOB1 mRNA. **b** MiR-363 mimic inhibited the luciferase activity of cells that transfected with the wild-type 3′-UTR of NOB1 mRNA. **c** The mRNA level of NOB1 was reduced in miR-363 overexpressed MG63 cells, whereas increased in miR-363 knocking down cells. **P < 0.01; ^#^*P* > 0.05; WT: wild type of ADAM9 3′UTR; MUT: mutant of ADAM9 3′UTR

TargetScan database was used for identifying the potential target genes of miR-363, and NOB1 gene was predicted as a potential target of miR-363. To test whether miR-363 directly binds to NOB1, the binding sequences at 217–223 on NOB1 3′-UTR were mutated from GUGCAAU to GACGGAA (Fig. 5a). Subsequently, the NOB1 wild-type or mutant 3′-UTR fragment was cloned into the pmirGlo vector, and then, we calculated the luciferase ability. As results, it was found that the miR-363 mimic inhibited (*P* < 0.05) the luciferase activity of cells transfected with the wild-type NOB1 3′-UTR, but it did not affect the luciferase activity of cells that transfected with the mutant 3′-UTR of NOB1 mRNA (*P* > 0.05) (Fig. 5b). What is more, the mRNA level of NOB1 was reduced in MG63 cells overexpressing miR-363 (*P* < 0.05), whereas the mRNA level of NOB1 was increased in cells knocking down miR-363 (*P* < 0.05) (Fig. 5c). In brief, the results showed that miR-363 could mediate the expression of NOB1 through binding to the 3′-UTR of NOB1 mRNA.

### NOB1 could partially reverse the roles of miR-363 in migration and invasion

**Fig. 6:**
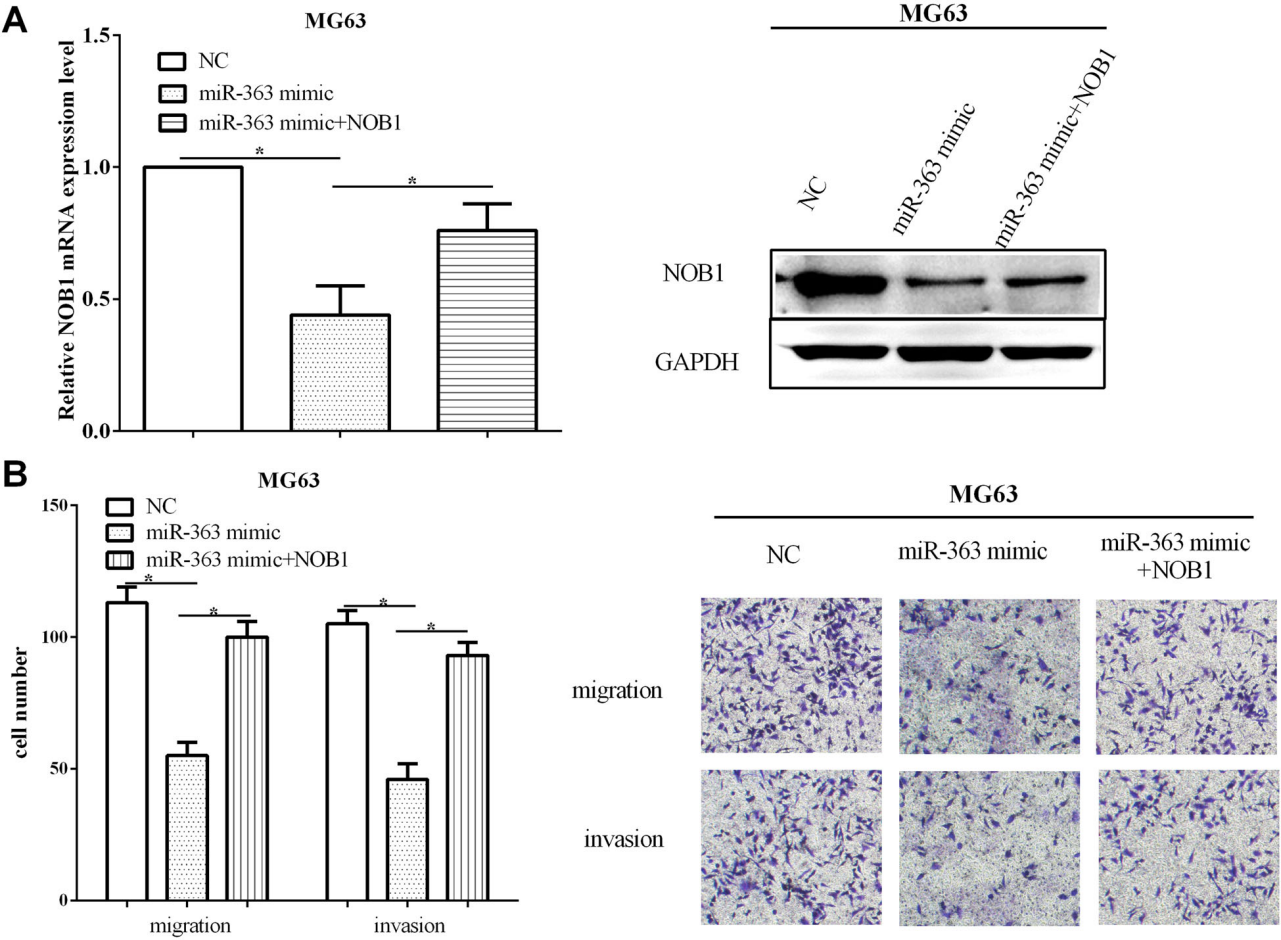
NOB1 could partially reverse the roles of miR-363 on cell migration and invasion. **a** NOB1 re-expressed vector pcDNA-NOB1 and the miR-363 mimic were co-transfected or only transfected with miR-363 mimic in MG63 cells. **b** Re-expression of NOB1 could recover the migratory and invasive abilities in miR-363 overexpressing MG63 cells. *P < 0.05; **P < 0.01; ***P < 0.001

Rescue experiment was performed to verify whether the impacts of miR-363 on cell migratory and invasive abilities were relied on NOB1. NOB1 overexpression vector pcDNA-NOB1 and the miR-363 mimic were co-transfected (*P* < 0.05) or only miR-363 mimic was transfected (*P* < 0.05) into MG63 cells, and qRT-PCR and Western blot were used to test whether the transfection was successful (Fig. 6a). Then, the capacities of cell migration and invasion were calculated by using transwell assay. NOB1 re-expression could recover the inhibitory effect of overexpressed miR-363 on migratory (*P* < 0.05) and invasive (*P* < 0.05) abilities of MG63 cells (Fig. 6b). Taken together, all the results demonstrated that NOB1 could partially reverse the roles of miR-363 in the migration and invasion of osteosarcoma cells.

### The expression of miR-363 and NOB1 related to survival of osteosarcoma

To explore the correlation between the expression of miR-363 and the clinicopathological characteristic of osteosarcoma, 49 patients were divided into high and low two groups according to the expression of miR-363. The relationship was examined between the expression of miR-363 and patients’ gender, age, tumor size, TNM stage, lymph node metastasis, metastasis, and the expression of NOB1, respectively. Chi-square test was performed to calculate the *P* value, and it was illuminated that the expression of miR-363 had a negative association with tumor size (*P* < 0.05), TNM stage (*P* < 0.05), lymph node metastasis (*P* < 0.05), and the expression of NOB1 in osteosarcoma tissues (*P* < 0.05) (Table 1).

**Table 1:** miR-363 expression and clinicopathological features in 49 osteosarcoma

Clinicopathological features	Cases (*n* = 49)	miR-363 expression		*P* value*
		23 high (%)	26 low (%)	
Gender				
Male	25	12 (48.0)	13 (52.0)	0.879
Female	24	11 (45.8)	13 (51.2)	
Age (years)				
≤ 18	21	13 (61.9)	8 (38.1)	0.124
> 18	28	10 (35.7)	18 (64.3)	
Tumor size (mm)				
≤ 5.0	22	14 (63.6)	8 (36.4)	0.035*
> 5.0	27	9 (33.3)	18 (66.7)	
TNM stage				
I–II	24	15 (62.5)	9 (37.5)	0.032*
III–IV	25	8 (32.0)	17 (68.0)	
Lymph node metastasis				
0–2	28	17 (60.7)	11 (39.3)	0.026*
> 2	21	6 (28.6)	15 (71.4)	
Metastasis				
Absent	23	14 (60.9)	9 (39.1)	0.066
Present	26	9 (34.6)	17 (65.4)	
NOB1				
Low expression	23	8 (34.8)	15 (65.2)	0.011*
High expression	26	15 (57.7)	11 (42.3)	

*P values are calculated with chi-square test

**Fig. 7:**
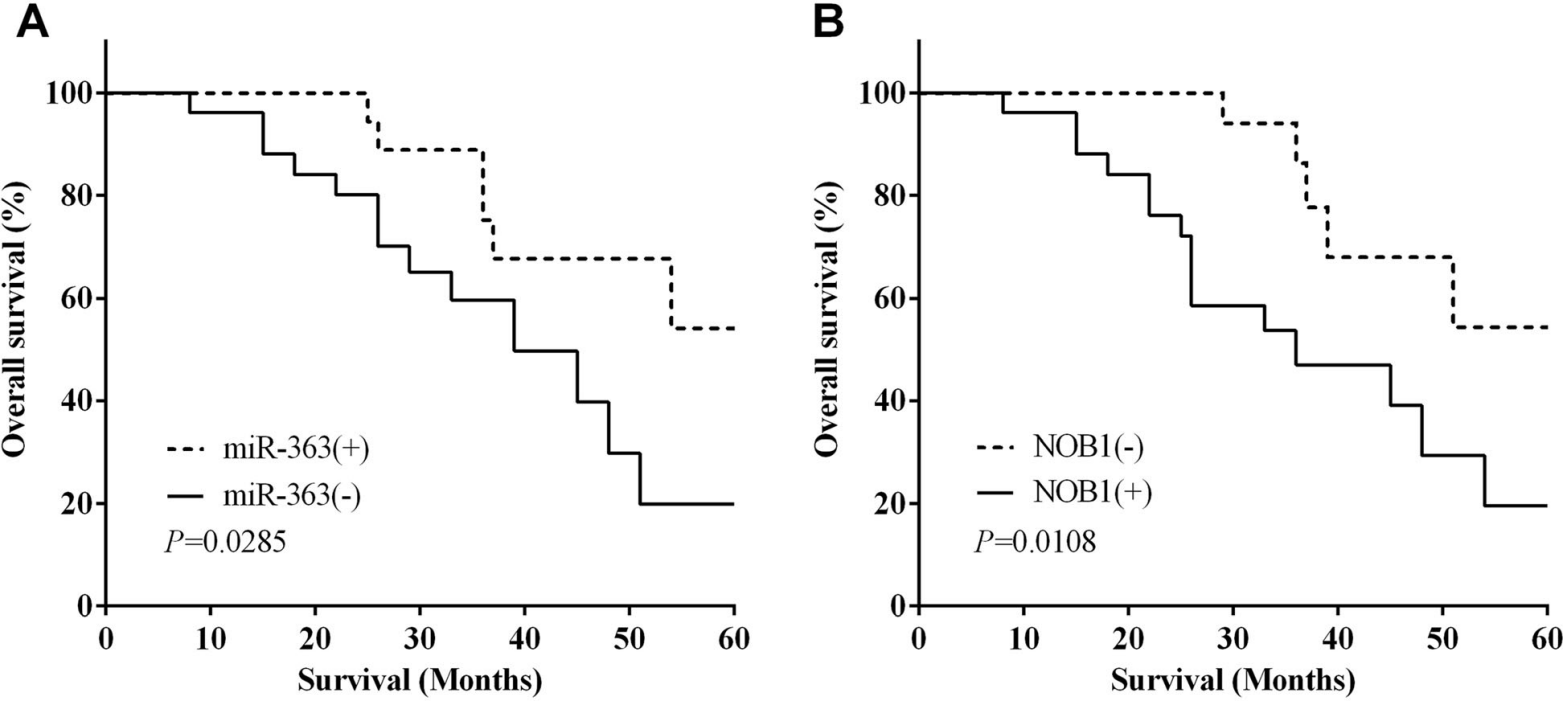
The expression of miR-363 and NOB1 related to survival of osteosarcoma. **a** The results demonstrated that the miR-363(−) group showed a lower 5-year overall survival than that of miR-363(+) group. **b** The 5-year overall survival was higher in the NOB1(−) group than in the NOB1(+) group

In addition, the log-rank (Mantel-Cox) test of Kaplan-Meier analysis was performed to calculate the relationship between the expression of miR-363 and NOB1 and the 5-year overall survival of osteosarcoma patients. The results demonstrated that the 5-year overall survival of miR-363(−) group was lower (log-rank *P* < 0.05) than that of the miR-363(+) group (Fig. 7a). On the contrary, the 5-year overall survival of the NOB1(−) group was higher than that of NOB1(+) group (*P* < 0.05) (Fig. 7b).

## Discussion

Osteosarcoma is a primary malignant bone tumor with a high metastasis rate and a shorter 5-year survival rate [2, 3]. Therefore, the study to investigate new biomarkers is essential to inhibit the metastasis of osteosarcoma. Increasing evidences demonstrated that miRNAs may play important functions in tumorigenesis and tumor progress [6, 7]. It was found that miR-363 was discovered to be downregulated and acted a tumor-suppressive role in various tumors [8–11]. In gastric cancer, miR-363 acted as a tumor suppressor and inhibited cell growth and migration [12]. A similar finding by Wang et al. demonstrated that miR-363 inhibited lung adenocarcinoma cell proliferation, colony formation, and tumor growth [13]. Our findings were consistent with all the reports that miR-363 was low expressed in osteosarcoma tissues and cell lines compared with the paracancerous tissues and matched normal cell line. What is more, overexpression of miR-363 could inhibit the proliferation, migration, and invasion, and induced cell apoptosis of osteosarcoma cell MG63. In contrast, eliminating miR-363 promoted the proliferative, migratory, and invasive abilities and inhibited the apoptosis ability of MG63 cells. In addition, we demonstrated that miR-363 inhibited the EMT of osteosarcoma cells MG63, which was consistent with the findings of Hu et al. [14]. Finally, the 5-year overall survival of the miR-363 low expression group was shorter than that of the high-expression group, and the expression of miR-363 was inversely related to tumor size, TNM stage, lymphatic metastasis, and the expression of NOB1. Our results were consistent with the findings of Yin et al. in hepatocellular carcinoma and osteosarcoma [25, 26].

NOB1 could cleave RNA substrate that contains the D site of pro-ribosomal RNA, thereby regulating protease functions and RNA metabolism [16]. NOB1 acted as an oncogene and was upregulated in a variety of cancers that include cervical cancer, gastric cancer, epithelial ovarian cancer, and non-small cell lung cancer [18–21]. Silencing of NOB1 could inhibit cell growth and metastasis of laryngeal cancer and colorectal cancer [27, 28]. We discovered similar findings that NOB1 was upregulated in osteosarcoma tissues and cell lines. The expression of NOB1 was negatively correlated with the expression of miR-363 in osteosarcoma tissues. In MG63 cells, knocking down NOB1 could suppress the migration and invasion, which has the same effects as that of overexpressing miR-363. Furthermore, silencing NOB1 inhibited the EMT of MG63 cell, which was the first time to propose the association between the expression of NOB1 and EMT and was the novelty of our study. Then, we verified that NOB1 was a target gene of miR-363 and was mediated by miR-363, which was consistent with the findings of Lin et al. in ovarian cancer [29]. In addition, NOB1 gene silencing can inhibit tumor growth by inducing apoptosis in human colorectal cancer and prostate cancer [30, 31]. Our result also proposed that upregulation of NOB1 predicted poor survival of osteosarcoma patients.

In conclusion, miR-363 suppressed the proliferation, migration, invasion, and EMT and induced cell apoptosis of MG63 cells. Interference of NOB1 could inhibit cell migration, invasion, and EMT, which is consistent with the effects of overexpressing miR-363. This study showed that miR-363 played a tumor-suppressive role in osteosarcoma, and the direct mechanism of NOB1 has revealed its potential mechanisms for proliferation, metastasis, and apoptosis. MiR-363 may be involved in the progression of osteosarcoma and represents a potential biomarker and target for the treatment of osteosarcoma.

## Data Availability

The datasets used and/or analyzed during the current study are available from the corresponding author on reasonable request.
